# Sex differences in the prevalence of high-risk oral and anal human papillomavirus infections among heterosexually active populations in Ibadan, Nigeria

**DOI:** 10.3389/frph.2025.1570984

**Published:** 2025-08-18

**Authors:** Imran Morhason-Bello, Yusuf Bello, Deborah Oke, Adekunle Daniel, Akinyele Adisa, Adeola Fowotade, Yinan Zheng, Joshua Akinyemi, Isaac Adewole, Miquel A. Pavon, Robert Murphy, Lifang Hou, Suzanna C. Francis, Deborah Watson-Jones

**Affiliations:** ^1^ Department of Obstetrics and Gynecology, Faculty of Clinical Sciences, College of Medicine, University of Ibadan, Ibadan, Nigeria; ^2^ Institute for Advanced Medical Research and Training, College of Medicine, University of Ibadan, Ibadan, Nigeria; ^3^ HPV Consortium, College of Medicine, University of Ibadan, Ibadan, Nigeria; ^4^ Department of Statistics, Faculty of Science, King AbdulAziz University, Jeddah, Saudi Arabia; ^5^ Department of Epidemiology and Medical Statistics, Faculty of Public Health, College of Medicine, University of Ibadan, Ibadan, Nigeria; ^6^ Department of Otolaryngology, Faculty of Clinical Sciences, College of Medicine, University of Ibadan, Ibadan, Nigeria; ^7^ Department of Oral Pathology, Faculty of Dentistry, College of Medicine, University of Ibadan, Ibadan, Nigeria; ^8^ Department of Medical Microbiology and Parasitology, College of Medicine, University of Ibadan, Ibadan, Nigeria; ^9^ Preventive Medicine Department, Cancer Epidemiology and Prevention, Northwestern University, Chicago, IL, United States; ^10^ Institute for Global Health, Northwestern University, Chicago, IL, United States; ^11^ Infection and Cancer Laboratory, Cancer Epidemiology Research Program, ICO, Bellvitge Biomedical Research Institute (IDIBELL), Barcelona, Spain; ^12^ International Statistics and Epidemiology Group, London School of Hygiene and Tropical Medicine, London, United Kingdom; ^13^ Department of Clinical Research, Faculty of Infectious and Tropical Diseases, London School of Hygiene and Tropical Medicine, London, United Kingdom; ^14^ Mwanza Intervention Trials Unit, National Institute for Medical Research, Mwanza, Tanzania

**Keywords:** oral, anal, high-risk, HPV, female, male, Nigeria

## Abstract

**Purpose:**

To determine sex differences in the prevalence of oral and anal high-risk HPV infections among heterosexually active males and females in Ibadan.

**Methods:**

This was a secondary analysis from the Sexual Behavior and HPV Infections in Nigerians in Ibadan (SHINI) study that involved sexually active males and females aged 18–45 years. After a face-to-face interview, samples were collected from the mouth, cervix, vulva, and anus by a sex-matched trained nurse. High-risk HPV (hrHPV) in oral or/and anal sites were primary outcome variables, profiled by Anyplex^TM^ II HPV28 assay. The participants' demographic characteristics, sexual behaviors, and social lifestyle were included as explanatory variables. The chi-square or Fisher exact test was used to investigate the association between the presence of hrHPV and the participants' characteristics. Multivariable logistic regression was conducted to test the association between the sex of participants and each of the primary outcome after adjusting for potential confounders. Statistical significance was set at *p* < 0.05.

**Results:**

A total of 625 females including 310 females in general population (FGP) and 315 female sex workers (FSWs) and 316 males were recruited. Oral hrHPV prevalence was higher among FGP and FSWs than among males (10.5% vs. 14.9% vs. 3.6%, *p* < 0.001), as was anal hrHPV prevalence (39.3% vs. 60.8% vs. 6.7%, *p* < 0.001). More FGP (7.5%) and FSWs (13.0%) than males (0.9%) had hrHPV at both oral and anal sites (*p* < 0.001). Males had significantly lower odds of oral hrHPV [adjusted odds ratio (aOR) = 0.43, 95% CI: 0.15–1.24] than FSWs and FGP [aOR = 1.70, 95% CI: 0.62–4.63]. The odds of anal hrHPV was significantly lower among males [aOR = 0.05, 95% CI: 0.03–0.08] compared to FSWs and FGP [aOR = 0.42, 95% CI: 0.30–0.58].

**Conclusion:**

Oral hrHPV, anal hrHPV, and hrHPV at both sites were more prevalent in females than in males in the heterosexually active population. These findings highlight the importance of developing targeted HPV prevention strategies that account for sex-specific risk factors and the potential biological underpinnings contributing to these disparities.

## 1 Introduction

Human papillomavirus (HPV) is commonly transmitted and acquired by unprotected sexual activity. In healthy populations, nearly 80% in the first year and 90% in the second year could clear HPV infections without any symptoms (1, 2). Generally, the persistence of high-risk HPV infections (hrHPV) is associated with the development of HPV-associated cancers in different anatomic sites. Several studies have shown increasing sex or gender disparities in the burden of oral and anal HPV-associated cancers in the United States and other high-income countries, after adjusting for race, social and lifestyle factors (3, 4).

Over the years, the burden of squamous cell oral and oropharyngeal cancers is rising in Europe and the United States generally with higher trends in heterosexually active men relative to women after adjusting for smoking, HIV infection, and other lifestyle factors (5, 6). However, most studies showed that the prevalence of anal cancer is higher in heterosexually active women than men after adjusting for HIV and other lifestyle factors (7). The relative differences in the burden of oral and oropharyngeal and anal cancers by sex were associated with sexual risk factors, including the role played by heterosexual males and females during oral and anal sex.

Oral and anal HPV infections are commonly reported in younger populations of males and females relative to older adults, irrespective of sexual orientation. The high prevalence of oral and anal HPV infections in young people was associated with increasing reports of oral and anal sexual behaviors in the general and key affected population, irrespective of sexual orientations (8). Many studies, particularly in high-income countries, are reporting differences in the prevalence and risk factors associated with HPV infections in males and females involved in heterosexual relationships (9–11). The suggested theory is that there could be differences between males and females in the vulnerability to acquisition and biological response to HPV infections at these anatomical sites separate from their history of sexual risk factors. For example, a review by Garutti et al., showed that men relative to women had a two to three times higher prevalence of oral HPV (10.1%–11.5% vs. 3.2%–3.6% respectively), five times as many incident oral high-risk HPV (7.3% vs. 1.4%), and six times higher of oral HPV-16 type (10.3% vs. 1.4%) (12). In the same review, many studies reported a higher prevalence of anal HPV in heterosexually active women than men.

It is important to understand the role of differences in the sex of people on the risk of acquiring oral and anal HPV infections within the population, particularly in Sub-Saharan Africa (SSA). Therefore, this study aimed to examine sex differences in the prevalence of and factors associated with oral and anal hrHPV. This information might help to unearth strategies that are sensitive to differences in the sex of people in the population to prevent the acquisition and transmission of oral and anal HPV infection.

## 2 Methods

### 2.1 Study design, population, and study site

This was a cross-sectional secondary data analysis of Sexual Behavior and HPV Infection in Nigerians in Ibadan (SHINI) study that recruited sexually active males and females in Mokola, Ibadan North Local Government Area as urban setting and Moniya/Sasa, Akinyele Local Government Area as rural/peri-urban setting. A detailed description of the study design and procedure has been described in previous publications (13–15).

The females' participants in this study comprised of Females in general population (FGP) and female sex workers (FSWs). FSWs are a key population in the context of sexually transmitted infections (STIs), particularly HPV because of their risky sexual behaviors and limited access to preventive healthcare services. FSWs were included in the SHINI study to allow for a more comprehensive assessment of the burden and distribution of cervical, vulva, oral and anal HPV infections among heterosexually active females in Ibadan. All the male participants were recruited within the general population in the two local government areas and none of them engaged in sex work.

### 2.2 Study procedures

#### 2.2.1 Sampling and enrolment of study participants

For males and FGP, a two-stage sampling procedure was used to choose eligible participants. We used the list of enumeration areas (EAs) prepared for the 2006 National Population Commission Census (16). The first stage of sampling involved a random selection of four EAs at Mokola and Moniya, as well as one EA at Sasa. Each residence received a personalized identification number. The research assistants visited houses in the selected EAs to conduct a census to list males and females aged 18–45 years. The second stage of sampling involved a selection of study participants using systematic random sampling from the sampling frame generated from house listing.

Participants were invited to the clinic individually to participate in the study. The gender-matched research assistants invited the selected participants to discuss the objectives of the study and gave a copy of the information leaflet to complement their discussion. Each participant was reminded at 72 and 24 h and on the day of participation for their clinic appointment.

At the clinic, a written informed consent was obtained. Afterwards, the research assistant conducted a 30 min face-to-face interview on sociodemographic, sexual behaviors, lifestyle characteristics, and knowledge about HPV, followed by clinical examination and collection of biological samples from the cervix, vulva, oral, and anal anatomical sites of female participants and from the penile, oral, and anal anatomical sites of male participants by sex-matched research nurses. Supplementary Figures S1, S2 present the enrollment flow charts for the FGP and male participants.

The list of brothels in the chosen LGAs was initially mapped by trained field workers for the FSWs. After interviewing the manager or the head of the FSWs, female research assistants compiled a list of eligible participants and assigned each one a unique number. All FSWs in brothels with ten or fewer eligible participants were recruited for the study, while eligible FSWs were randomly selected from brothels with more than ten eligible participants. Female research assistants then visited each brothel, distributed information leaflets, and explained the study's objectives to participants individually and in groups in their rooms. Each participant's bedroom served as the location for the face-to-face interview and sample collection, during which data on sociodemographic characteristics, sexual behaviors, lifestyle, and knowledge about HPV were collected. Supplementary Figure S3 presents the enrollment flowchart for the FSWs.

All participants consented and had HIV testing (RDT) in accordance with the National Guidelines for HIV Prevention, Treatment, and Care published by the Federal Ministry of Health in Nigeria (17). A detailed description of the survey completion rates, oral and anal sample collection, transport, and storage has been published (13–15). Participants were given toiletries and transport money for their participation.

The HPV genotyping was performed using Anyplex^™^ II HPV28 assay (Seegene, Seoul, South Korea) in accordance with the manufacturer's guidelines at the Catalan Institute of Oncology in Spain. The team extracted the DNA samples from oral and anal samples using Maxwell® 16 LEV Blood DNA kit (Promega Corp., Madison, WI, USA) and Maxwell 16 Buccal swab LEV DNA Purification kit (Promega Corp., Madison, WI, USA), respectively.

### 2.3 Ethical approval

The study was conducted in accordance with the Declaration of Helsinki and approved by the Ethics Committee of the London School of Hygiene and Tropical Medicine, London (LSHTM 9736–3); the University of Ibadan/the University College Hospital, Ibadan (UI/EC/16/005); and the Oyo State Government (AD13/479/712) in Nigeria.

### 2.4 Data entry and analysis

The data was analyzed with STATA 15.0 (Stata 2019. Statistical Software: Release 15. College Station, TX: StataCorp LLC) software. The descriptive analysis of selected sociodemographic, sexual behaviors, social and lifestyle, and biological characteristics was performed. Categorical variables were summarized using frequency and percentage, while mean and standard deviation or median and interquartile range were used to summarize continuous variables as appropriate. The primary outcome variable was any hrHPV (HPV -16, -18, -31, -33, -35, -39, -45, -51, -52, -56, -58, -59, -66, -68) in the oral site, anal site, and both sites. In this analysis, the primary exploratory variable was the sex of participants (males vs. females), while other sociodemographic, sexual behavior, social and lifestyle, and biological characteristics were included as secondary explanatory variables. The association between oral or/and anal hrHPV and sex of participants was assessed with crude logistic regression model. The final adjusted logistic regression model for oral or/and anal hrHPV included additional secondary explanatory variables covering sociodemographic, sexual behavior, social and lifestyle, and biological characteristics as the covariates. These secondary explanatory variables were included based on theoretical considerations drawn from existing literature, expert knowledge, and relevant conceptual frameworks. Listwise deletion was applied in the tests of association and multivariable models to handle missing responses on any of the variables.

To account for multiple hypotheses testing in the multivariable models, Hommel's correction was applied to the *p*-values of all secondary explanatory variables, excluding the primary variable (sex), to differentiate robust associations from those possibly arising by chance (18). Hommel's method was selected because it controls the family-wise error rate and has been recognized for its improved balance between type I error control and statistical power than traditional Bonferroni or Benjamini-Hochberg correction (19, 20). A total of 12 independent hypotheses were tested for oral and anal hrHPV, respectively, and 13 independent hypotheses for hrHPV in both anatomical sites, depending on the number of covariates included in each multivariable model. The adjusted *p*-values are presented in the supplementary multivariable logistics regression table. Statistical significance was set at *p* < 0.05, both before and after adjustment.

Post-hoc power analysis was conducted to assess the adequacy of the sample sizes for detecting sex differences in hrHPV prevalence across the three groups (males, FGP, and FSWs). Power was computed based on the observed prevalence and sample distributions using STATA 15.0.

## 3 Results

The data of 625 females, of whom 315 were FSWs, and 316 males were analyzed. In total, 10.5% of FGP, 14.9% of FSWs, and 3.6% of males had at least one hrHPV infection in the oral cavity (Table 1). Regardless of the female subgroups, the overall prevalence of oral hrHPV among females was 12.7% (Supplementary Table S1). Table 2 shows sex differences in the prevalence of oral hrHPV. The prevalence of oral hrHPV was significantly higher in urban than rural settings in males (*p* = 0.039) and FGP (*p* = 0.021). None of the other sociodemographic characteristics and sexual behavioral characteristics was significantly associated with oral hrHPV among participants. None of the participants with oral hrHPV (male or female) had ever used a condom or any form of protection while engaging in oral sex (received or given) with their partners.

**Table 1:** Prevalence of oral, anal and both sites high-risk HPV by sex.

Variable	Sex			*p*-value
	Female		Male	
			*n* (%)	
	FGP	FSW		
	*n* (col %)	*n* (col %)		
Oral hrHPV				<0.001
No	256 (89.5)	240 (85.1)	297 (96.4)	
Yes	30 (10.5)	42 (14.9)	11 (3.6)	
Anal hrHPV				<0.001
No	184 (60.7)	122 (39.2)	223 (93.3)	
Yes	119 (39.3)	189 (60.8)	16 (6.7)	
Oral and Anal hrHPV				<0.001
No	259 (92.5)	242 (87.1)	229 (99.1)	
Yes	21 (7.5)	36 (13.0)	2 (0.9)	

**Table 2:** Participant characteristics and prevalence of oral high-risk HPV by sex.

Explanatory Variable	FGP^1^		*p*-value	FSW^2^		*p*-value	Male^3^		*p*-value
	Oral hrHPV			Oral hrHPV			Oral hrHPV		
	Yes (*N* = 30)	No (*N* = 256)		Yes (*N* = 42)	No (*N* = 240)		Yes (*N* = 11)	No (*N* = 297)	
	*n* (col %)	*n* (col %)		*n* (col %)	*n* (col %)		*n* (col %)	*n* (col %)	
Socio-demographic characteristics									
Age			0.965			0.143			0.734
18–24 years	12 (40.0)	99 (38.7)		9 (21.4)	37 (15.4)		4 (36.4)	131 (44.1)	
25–34 Years	9 (30.0)	83 (32.4)		26 (61.9)	128 (53.3)		3 (27.3)	90 (30.3)	
35–45 Years	9 (30.0)	74 (28.9)		7 (16.7)	75 (31.3)		4 (36.4)	76 (25.6)	
Education			0.933			0.077			>0.999
None	0	4 (1.6)		6 (14.3)	13 (5.4)		0	1 (0.3)	
Primary	5 (16.7)	48 (18.8)		6 (14.3)	54 (22.5)		0	17 (5.7)	
Secondary	19 (63.3)	146 (57.0)		24 (57.1)	153 (63.8)		9 (81.8)	210 (70.7)	
Tertiary	6 (20.0)	58 (22.7)		6 (14.3)	20 (8.3)		2 (18.2)	69 (23.2)	
Occupation			0.911			0.872			0.858
No current paid job	6 (20.0)	44 (17.2)		0	0		3 (27.3)	55 (18.5)	
Unskilled	2 (6.7)	15 (5.9)		32 (76.2)	175 (72.9)		0	8 (2.7)	
Semi-Skilled	21 (70.0)	181 (70.7)		10 (23.8)	64 (26.7)		7 (63.6)	191 (64.3)	
Skilled	1 (3.3)	16 (6.3)		0	1 (0.4)		1 (9.1)	43 (14.5)	
Monthly Income			0.927			0.946			0.862
No income	3 (10.0)	31 (12.1)		0	3 (1.3)		1 (9.1)	34 (11.5)	
1–10,000 N (1–28USD)	11 (36.7)	101 (39.5)		2 (4.8)	12 (5.0)		3 (27.3)	68 (22.9)	
10,001–20,000 N (>28–56USD)	8 (26.7)	69 (27.0)		12 (28.6)	61 (25.4)		1 (9.1)	64 (21.6)	
>20,000 N (>56USD)	8 (26.7)	55 (21.5)		28 (66.7)	164 (68.3)		6 (54.6)	131 (44.1)	
Marital Status			0.838			0.294			0.294
Single	8 (26.7)	71 (27.7)		20 (47.6)	129 (53.8)		6 (54.6)	189 (63.6)	
Married	20 (66.7)	172 (67.2)		0	10 (4.2)		4 (36.4)	100 (33.7)	
Divorced/Widow	2 (6.7)	13 (5.1)		22 (52.4)	101 (42.1)		1 (9.1)	8 (2.7)	
Partner has another sexual partner [(FGP, *N* = 275), (Male, *N* = 279)]			0.668			0.315			0.542
Don't know	5 (17.2)	36 (14.6)		24 (57.1)	116 (48.3)		3 (37.5)	75 (27.7)	
No	15 (51.7)	148 (60.2)		4 (9.5)	45 (18.8)		5 (62.5)	154 (56.8)	
Yes	9 (31.0)	62 (25.2)		14 (33.3)	79 (32.9)		0	42 (15.5)	
Study Setting			0.021			0.156			0.039
Urban	21 (70.0)	122 (47.7)		35 (83.3)	219 (91.3)		9 (81.8)	150 (50.5)	
Rural	9 (30.0)	134 (52.3)		7 (16.7)	21 (8.8)		2 (18.2)	147 (49.5)	
Sexual Behavioral Characteristics									
Age at first gave oral sex [mean (SD)]	32.3 (7.2)	25.5 (1.1)	0.090	22 (1.3)	26.6 (1.2)	0.098	25.8 (7.7)	-	-
Age at first received oral sex [mean (SD)]	27.6 (5.0)	24.2 (1.1)	0.301	27.6 (1.2)	27.4 (0.6)	0.913	26 (1.00)	24.1 (0.7)	0.705
Ever given oral sex			0.548			0.367			0.373
Yes	4 (13.3)	27 (10.6)		10 (23.8)	43 (17.9)		0	43 (14.5)	
No	26 (86.7)	229 (89.5)		32 (76.2)	197 (82.1)		11 (100)	254 (85.5)	
Ever received oral sex			0.364			0.245			0.514
Yes	5 (16.7)	28 (10.9)		21 (50.0)	97 (40.4)		2 (18.2)	99 (33.3)	
No	25 (83.3)	228 (89.1)		21 (50.0)	143 (59.6)		9 (81.8)	198 (66.7)	
Number of oral sex partners [median (IQR)]	1 (0)	1 (0)		1 (1)	1 (1)		1 (1)		
Condom/barrier use during last oral sex [(FGP, *N* = 31), (FSWs, *N* = 53), (Male, *N* = 43)]			>0.999			0.323			-
Yes	0 (0)	1 (3.7)		0	7 (16.3)		0	3 (7.0)	
No	4 (100)	26 (96.3)		10 (100)	36 (83.7)		0	40 (93.0)	
Social and Lifestyle Characteristics									
Ever drank alcohol			0.403			0.542			0.528
Yes	20 (66.7)	189 (73.8)		30 (71.4)	182 (75.8)		6 (54.6)	193 (65.0)	
No	10 (33.3)	67 (26.2)		12 (28.6)	58 (24.2)		5 (45.5)	104 (35.0)	
Ever taken any illicit drugs			-						>0.999
Yes	0	0		0	0		0	3 (1.0)	
No	30 (100)	256 (100)		42 (100)	240 (100)		11 (100)	294 (99.0)	
Ever smoked tobacco or cigarette			>0.999			0.394			0.190
Yes	0	3 (1.2)		15 (35.7)	70 (29.2)		1 (9.1)	86 (29.0)	
No	30 (100)	253 (98.8)		27 (64.3)	170 (70.8)		10 (90.9)	211 (71.0)	
Biological Characteristics									
Ever had any STI			>0.999			0.617			>0.999
No	26 (86.7)	219 (85.6)		35 (83.3)	207 (86.3)		11 (100)	295 (99.3)	
Yes	4 (13.3)	37 (14.5)		7 (16.7)	33 (13.8)		0	2 (0.7)	
Diagnosed of HIV			**0** **.** **001**			**0** **.** **042**			-
Yes	4 (13.3)	2 (0.8)		10 (23.8)	29 (12.1)		0	0	
No	26 (86.7)	254 (99.2)		32 (76.2)	211 (87.9)		11 (100)	297 (100)	

hrHPV-16, 18, 31, 33, 35, 39, 45, 51, 52, 56, 58, 59, 66, 68; 1–24 participants with invalid sample; 2–33 participants with invalid sample; 3–8 participants with invalid sample.

The bold values in the table indicate significant *p*-value.

**Figure 1:**
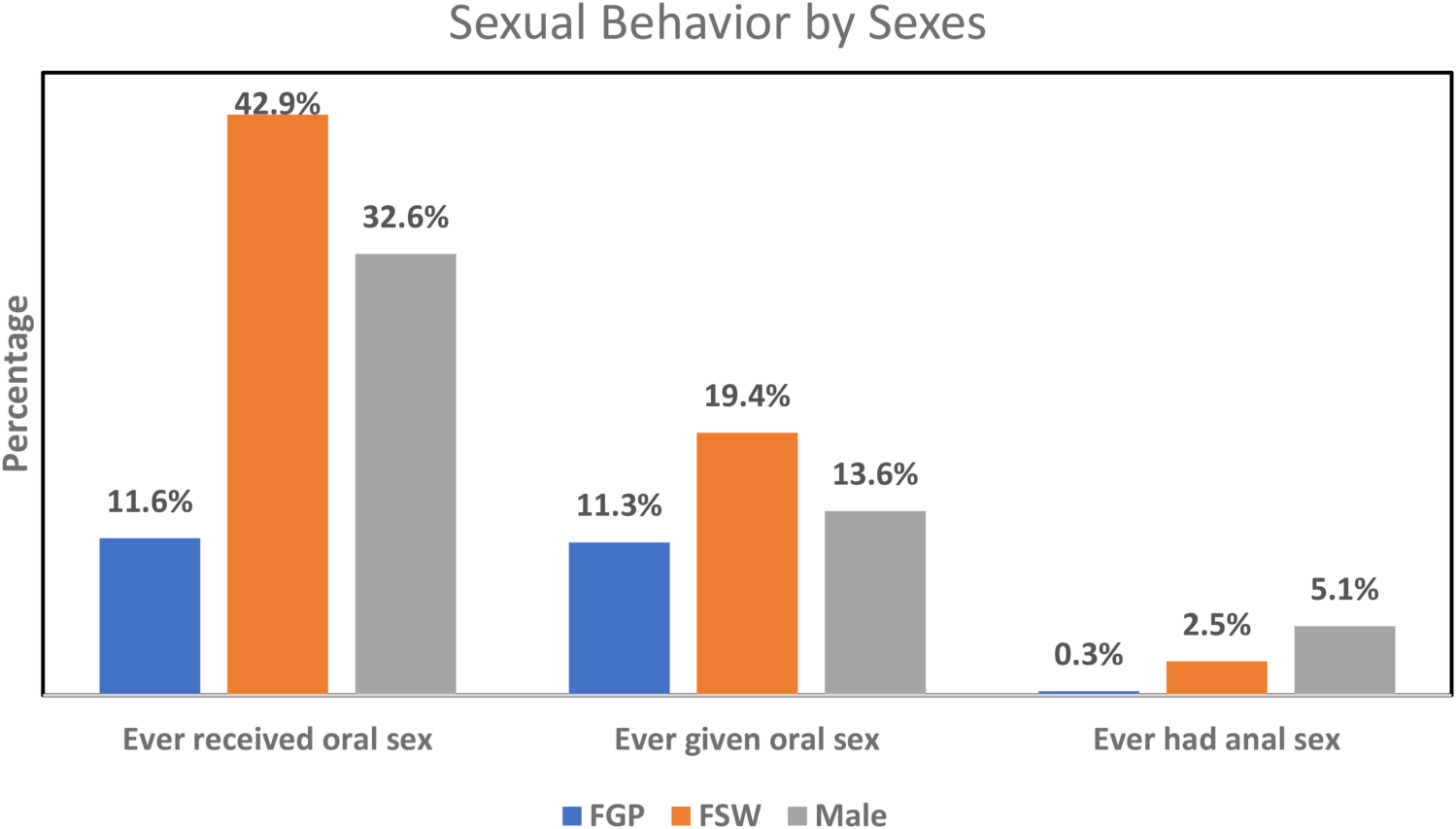
Pattern of oral and anal sexual behaviors among males and females.

The mean age at oral sex debut among FGP, FSWs, and males was not significantly different between those with oral hrHPV and those without. Oral sexual behaviors were more prevalent among FSWs than among FGP and males, while such behaviors were more commonly practiced among males than FGP (Figure 1). None of the male participants with oral hrHPV had ever given oral sex while 13.3% of FGP and 23.8% of FSWs with oral hrHPV had given oral sex to their sexual partners. However, 18.2% of male participants with oral hrHPV had received oral sex from their sexual partners.

Regarding social and lifestyle characteristics, the prevalence of oral hrHPV was higher among FGP (66.7%), FSWs (71.4%), and males (54.6%) who consumed alcohol compared to those who did not. However, none of the social or lifestyle characteristics was significantly associated with oral hrHPV among the participants. HIV-positive FGP and FSWs had significantly higher prevalence of oral hrHPV compared to HIV-negative FGP and FSWs. None of the male participants, regardless of oral hrHPV status, was HIV positive (Table 2).

Overall, the prevalence of anal hrHPV was significantly higher among females (50.2%) than among males (6.7%) (Supplementary Table S1). A higher percentage of FSWs (60.8%) engaged in anal sex compared to FGP (39.3%), while anal sex was more prevalent among FGP than among males (6.7%). Education (*p* = 0.009) and occupation (*p* = 0.042) were associated with anal hrHPV among FGP, while age (*p* = 0.006) was associated with anal sex among FSWs. None of these factors was associated with anal hrHPV among males. The prevalence of anal hrHPV was highest among FGP whose current sexual partner had no other sexual partners (57.1%) and lowest among those who were unaware of their partner's sexual relationships with other women (17.0%). The prevalence of anal hrHPV was higher in participants living in urban settings compared to those in peri-urban or rural areas. The average age at first anal sex was higher among FGP with anal hrHPV (24.0 ± 2.6 years) compared to those without (21.7 ± 0.9 years). Alcohol consumption (*p* = 0.003) and HIV status (*p* = 0.043) were significantly associated with anal hrHPV among FGP (Table 3). Figure 1 gives a graphical representation of anal sexual behaviors by sex.

**Table 3:** Participant characteristics and prevalence of anal high-risk HPV by sex.

Explanatory Variable	FGP^1^		*p*-value	FSW^2^		*p*-value	Male^3^		*p*-value
	Anal hrHPV			Anal hrHPV			Anal hrHPV		
	Yes (*N* = 119)	No (*N* = 184)		Yes (*N* = 189)	No (*N* = 122)		Yes (*N* = 16)	No (*N* = 223)	
				*n* (col %)	*n* (col %)				
	*n* (col %)	*n* (col %)					*n* (col %)	*n* (col %)	
Socio-demographic Characteristics									
Age			0.243			**0** **.** **006**			0.760
18–24 years	53 (44.5)	67 (36.4)		44 (23.3)	11 (9.0)		6 (37.5)	99 (44.4)	
25–34 Years	38 (31.9)	59 (32.1)		97 (51.3)	74 (60.7)		6 (37.5)	67 (30.0)	
35–45 Years	28 (23.5)	58 (31.5)		48 (25.4)	37 (30.3)		4 (25.0)	57 (25.6)	
Education			**0** **.** **009**			0.359			0.908
None	1 (0.8)	4 (2.2)		11 (5.8)	10 (8.2)		0	1 (0.5)	
Primary	12 (10.1)	44 (23.9)		44 (23.3)	21 (17.2)		0	12 (5.4)	
Secondary	77 (64.7)	94 (51.1)		116 (61.4)	83 (68.0)		13 (81.3)	159 (71.3)	
Tertiary	29 (24.4)	42 (22.8)		18 (9.5)	8 (6.6)		3 (18.8)	51 (22.9)	
Occupation			**0** **.** **042**			0.815			0.432
No current paid job	30 (25.2)	24 (13.0)		139 (73.5)	87 (71.3)		1 (6.3)	46 (20.6)	
Unskilled	5 (4.2)	13 (7.1)		0	0		0	3 (1.4)	
Semi-Skilled	78 (65.6)	133 (72.3)		49 (25.9)	35 (28.7)		13 (81.3)	139 (62.3)	
Skilled	6 (5.0)	14 (7.6)		1 (0.5)	0		2 (12.5)	35 (15.7)	
Monthly Income			0.066			0.311			0.209
No income	17 (14.3)	18 (9.8)		1 (0.5)	2 (1.6)		1 (6.3)	26 (11.7)	
1–10,000 N (1–28USD)	49 (41.2)	73 (39.7)		8 (4.2)	7 (5.7)		6 (37.5)	45 (20.2)	
10,001–20,000 N (> 28–56USD)	23 (19.3)	59 (32.1)		44 (23.3)	36 (29.5)		1 (6.3)	53 (23.8)	
>20,000 N (> 56USD)	30 (25.2)	34 (18.5)		136 (72.0)	77 (63.1)		8 (50.0)	99 (44.4)	
Marital Status			0.431			0.915			0.177
Single	36 (30.3)	48 (26.1)		100 (52.9)	64 (52.5)		12 (75.0)	141 (63.2)	
Married	75 (63.0)	128 (69.6)		8 (4.2)	4 (3.3)		3 (18.8)	78 (35.0)	
Divorced/Widow	8 (6.7)	8 (4.4)		81 (42.9)	54 (44.3)		1 (6.3)	4 (1.8)	
Partner has another sexual partner [(FGP, *N* = 291), (Male, *N* = 216)]			0.526			0.864			0.492
Don't know	19 (17.0)	22 (12.3)		93 (49.2)	60 (49.2)		6 (40.0)	53 (26.4)	
No	64 (57.1)	110 (61.5)		35 (18.5)	20 (16.4)		8 (53.3)	115 (57.2)	
Yes	29 (25.9)	47 (26.3)		61 (32.3)	42 (34.4)		1 (6.7)	33 (16.4)	
Study Setting			0.063			0.536			0.087
Urban	68 (57.1)	85 (46.2)		170 (90.0)	107 (87.7)		11 (68.8)	104 (46.6)	
Rural	51 (42.9)	99 (53.8)		19 (10.0)	15 (12.3)		5 (31.3)	119 (53.4)	
Sexual Behavioral Characteristics									
Age at first anal sex [mean (SD)]	24 (2.6)	21.7 (0.9)	0.557	24.0 (2.6)	21.5 (1.5)	0.615	19.6 (4.3)	-	-
Ever had anal sex			> 0.999			0.488			0.530
Yes	0	1 (0.5)		6 (3.2)	2 (1.6)		0	9 (4.0)	
No	119 (100)	183 (99.5)		183 (96.8)	120 (98.4)		16 (100)	214 (96.0)	
Number of anal sex partners [median (IQR)]	1 (0)	1 (1)		-	2 (2)				
Condom/barrier use during last anal sex [(FGP, *N* = 1), (FSW, *N* = 8), (Male, *N* = 9)]			-			0.464			-
Yes	0	0		3 (50.0)	2 (100)		1 (11.1)	0	
No	0	1 (100)		3 (50.0)	0		8 (88.9)	0	
Social and Lifestyle Characteristics									
Ever drank alcohol			**0** **.** **003**			0.707			0.608
Yes	76 (63.9)	146 (79.4)		143 (75.7)	90 (73.8)		11 (68.8)	139 (62.3)	
No	43 (36.1)	38 (20.7)		46 (24.3)	32 (26.2)		5 (31.3)	84 (37.7)	
Ever taken any illicit drugs			-			-			>0.999
Yes	0	0		0	0		0	2 (0.9)	
No	119 (100)	184 (100)		189 (100)	122 (100)		16 (100)	221 (99.1)	
Ever smoked tobacco or cigarette			0.216			0.146			0.360
Yes	4 (3.4)	2 (1.1)		66 (34.9)	33 (27.1)		6 (37.5)	60 (26.9)	
No	115 (96.6)	182 (98.9)		123 (65.1)	89 (73.0)		10 (62.5)	163 (73.1)	
Biological Characteristics									
Ever had any STI			0.061			0.269			0.704
No	97 (81.5)	164 (89.1)		165 (87.3)	101 (82.8)		16 (100)	221 (99.1)	
Yes	22 (18.5)	20 (10.9)		24 (12.7)	21 (17.2)		0	2 (0.9)	
Diagnosed of HIV			**0** **.** **043**			**0** **.** **001**			-
Yes	6 (5.0)	2 (1.1)		35 (18.5)	7 (5.7)		0	0	
No	113 (95.0)	182 (98.9)		154 (81.5)	115 (94.3)		16 (100)	223 (100)	

hrHPV-16, 18, 31, 33, 35, 39, 45, 51, 52, 56, 58, 59, 66, 68; 1–7 participants with invalid sample, 2–4 participants with invalid sample; 3–77 participants with invalid sample.

The bold values in the table indicate significant *p*-value.

More FGP (7.5%) and FSWs (13.0%) had hrHPV at both oral and anal sites compared to males (0.9%) (*p* < 0.001) (Table 1). Among FGP, only HIV status was associated with hrHPV at both oral and anal sites. Among FSWs, education (*p* = 0.015), history of anal sex (*p* = 0.011), and HIV status (*p* = 0.011) were significantly associated with hrHPV at both sites. Regardless of female subgroup, level of education (*p* = 0.032), marital status (*p* = 0.034), having a partner with other sexual partners (*p* = 0.036), history of anal sex (*p* < 0.005), tobacco or cigarette use (*p* = 0.016), and HIV status (*p* < 0.001) were all significantly associated with hrHPV at both oral and anal sites (Supplementary Table S3). In contrast, none of these factors were significantly associated with hrHPV at both sites among males (Table 4).

**Table 4:** Participant characteristics and prevalence of high-risk HPV in both oral and anal sites by sex.

Explanatory Variable	FGP^1^		*p*-value	FSW^2^		*p*-value	Male^3^		*p*-value
	Oral and Anal hrHPV			Oral and Anal hrHPV			Oral and Anal hrHPV		
	Yes (*N* = 21)	No (*N* = 259)		Yes (*N* = 36)	No (*N* = 242)		Yes (*N* = 2)	No (*N* = 229)	
	*n* (col %)	*n* (col %)		*n* (col %)	*n* (col %)		*n* (col %)	*n* (col %)	
Socio-demographic Characteristics									
Age			0.652			0.107			0.735
18–24 years	10 (47.6)	100 (38.6)		8 (22.2)	38 (15.7)		1 (50.0)	101 (44.1)	
25–34 Years	5 (23.8)	84 (32.4)		23 (63.9)	130 (53.7)		0	69 (30.1)	
35–45 Years	6 (28.6)	75 (29.0)		5 (13.9)	74 (30.6)		1 (50.0)	59 (25.8)	
Education			0.643			**0** **.** **015**			>0.999
None	0	3 (1.2)		6 (16.7)	13 (5.4)		0	1 (0.4)	
Primary	2 (9.5)	51 (19.7)		5 (13.9)	55 (22.7)		0	12 (5.2)	
Secondary	14 (66.7)	146 (56.4)		19 (52.8)	156 (64.5)		2 (100)	164 (71.6)	
Tertiary	5 (23.8)	59 (22.8)		6 (16.7)	18 (7.4)		0	52 (22.7)	
Occupation			0.937			0.727			0.148
No current paid job	5 (23.8)	45 (17.4)		28 (77.8)	176 (72.7)		1 (50.0)	44 (19.2)	
Unskilled	1 (4.8)	16 (6.2)		0	0		0	3 (1.3)	
Semi-Skilled	14 (66.7)	182 (70.3)		8 (22.2)	65 (26.9)		0	147 (64.2)	
Skilled	1 (4.8)	16 (6.2)		0	1 (0.4)		1 (50.0)	35 (15.3)	
Monthly Income			0.147			0.906			0.407
No income	3 (14.3)	31 (12.0)		0	3 (1.2)		1 (50.0)	25 (10.9)	
1–10,000 N (1–28USD)	4 (19.1)	105 (40.5)		2 (5.6)	12 (5.0)		0	51 (22.3)	
10,001–20,000 N (>28–56USD)	6 (28.6)	68 (26.3)		10 (27.8)	62 (25.6)		0	51 (22.3)	
>20,000 N (> 56USD)	8 (38.1)	55 (21.2)		24 (66.7)	165 (68.2)		1 (50.0)	102 (44.5)	
Marital Status			0.528			0.361			0.568
Single	8 (38.1)	71 (27.4)		17 (47.2)	130 (53.7)		2/2 (100)	147 (64.2)	
Married	12 (57.1)	174 (67.2)		0	10 (4.1)		0	77 (33.6)	
Divorced/Widow	1 (4.8)	14 (5.4)		19 (52.8)	102 (42.2)		0	5 (2.2)	
Partner has another sexual partner [(FGP, *N* = 269), (FSW, *N* = 278) (Male, *N* = 208)]			0.433			0.257			0.094
Don't know	5 (25.0)	35 (14.1)		21 (58.3)	116 (47.9)		2/2 (100)	53 (25.7)	
No	11 (55.0)	148 (59.4)		3 (8.3)	46 (19.0)		0	120 (58.3)	
Yes	4 (20.0)	66 (26.5)		12 (33.3)	80 (33.1)		0	33 (16.0)	
Study Setting			0.112			0.228			0.230
Urban	14 (66.7)	126 (48.7)		30 (83.3)	220 (90.9)		2 (100)	109 (47.6)	
Rural	7 (33.3)	133 (51.4)		6 (16.7)	22 (9.1)		0	120 (52.4)	
Sexual Behaviors Characteristics									
**Age at first gave oral sex** [mean (SD)]	28 (10)	26.3 (1.2)	0.730	22 (1.3)	26.8 (1.2)	0.091	-	-	-
**Age at first received oral sex** [mean (SD)]	24.3 (6.8)	24.9 (1.2)	0.895	26.9 (1.2)	27.5 (0.6)	0.709	-	-	-
Ever given oral sex			0.480			0.135			>0.999
Yes	3 (14.3)	27 (10.4)		10 (27.8)	42 (17.4)		0	34 (14.9)	
No	18 (85.7)	232 (89.6)		26 (72.2)	200 (82.6)		2 (100)	195 (85.2)	
Ever received oral sex			0.718			0.260			0.548
Yes	3 (14.3)	29 (11.2)		18 (50.0)	97 (40.1)		0	79 (34.5)	
No	18 (85.7)	230 (88.8)		18 (50.0)	145 (59.9)		2 (100)	150 (65.5)	
Number of oral sex partners [median (IQR)]	1 (0)	1 (0)		1 (1)	1 (1)		-	-	-
Ever had anal sex			-			**0** **.** **011**			>0.999
Yes	0	0		4 (11.1)	4 (1.7)		0	8 (3.5)	
No	21 (100)	259 (100)		32 (88.9)	238 (98.4)		2 (100)	221 (96.5)	
**Age at first anal sex** [mean (SD)]	-	-	-	22.8 (1.7)	24.0 (3.8)	0.774	-	-	-
Number of anal sex partners [median (IQR)]	-	-	-	1 (2)	1 (0)		-	-	-
Condom/barrier use during last oral sex [(FGP, *N* = 30), (FSW, *N* = 52), (Male, *N* = 34)]			>0.999			0.322			-
Yes	0	1 (3.7)		0	7 (16.7)		-	3 (8.8)	
No	3 (100)	26 (96.3)		10 (100)	35 (83.3)		-	31 (91.2)	
Social and Lifestyle Characteristics									
Ever drank alcohol			0.224			0.684			>0.999
Yes	13 (61.9)	192 (74.1)		26 (72.2)	182 (75.2)		1 (50.0)	143 (62.5)	
No	8 (38.1)	67 (25.9)		10 (27.8)	60 (24.8)		1 (50.0)	86 (37.6)	
Ever taken any illicit drugs			-			-			>0.999
Yes	0	0		0	0		0	2 (0.9)	
No	21 (100)	259 (100)		36 (100)	242 (100)		2 (100)	227 (99.1)	
Ever smoked tobacco or cigarette			>0.999			0.097			>0.999
Yes	0	3 (1.2)		15 (41.7)	68 (28.1)		0	64 (28.0)	
No	21 (100)	256 (98.8)		21 (58.3)	174 (71.9)		2 (100)	165 (72.1)	
Biological Characteristics									
Ever had any STI			>0.999			0.625			>0.999
No	18 (85.7)	222 (85.7)		30 (83.3)	209 (86.4)		2 (100)	227 (99.1)	
Yes	3 (14.3)	37 (14.3)		6 (16.7)	33 (13.6)		0	2 (0.9)	
Diagnosed of HIV			**<0.001**			**0** **.** **011**			-
Yes	4 (19.1)	2 (0.8)		10 (27.8)	29 (12.0)		0	0	
No	17 (80.9)	257 (99.2)		26 (72.2)	213 (88.0)		2 (100)	229 (100)	

hrHPV-16,18,31,33,35,39,45,51,52,56,58,59,66,68; 1–30 participants with invalid sample; 2–37 participants with invalid sample; 3–85 participants with invalid sample.

The bold values in the table indicate significant *p*-value.

Post-hoc power analysis confirmed that the sample size was sufficient to detect statistically significant differences in hrHPV prevalence between males and FGP, and between males and FSWs at the oral site (90.99% and 99.76%, respectively), anal site (>99.99% for both comparisons), and both sites (96.10% and 99.98%, respectively) (Supplementary Table S5). The estimated power for these comparisons indicated a very high likelihood of detecting true differences at the observed prevalence rates and significance level.

Table 5 presents the crude and adjusted odds ratios of factors associated with oral and/or anal hrHPV. Sex was significantly associated with the odds of having oral hrHPV in both the crude and adjusted models. In the crude model, the odds of oral hrHPV was higher among FGP [crude odds ratio (cOR) = 0.67, 95% CI: 0.41–1.10] than among males (cOR = 0.21, 95% CI: 0.11–0.42). Both FGP and males had lower odds of oral hrHPV compared to FSWs. After adjusting for participants' sociodemographic, lifestyle, and biological characteristics, FGP had significantly higher odds of oral hrHPV [Adjusted Odds Ratio (aOR) = 1.70, 95% CI: 0.62–4.63] compared to FSWs, while males had lower odds (aOR = 0.43, 95% CI: 0.15–1.24). The odds of oral hrHPV was 3.37 times higher among HIV-positive participants (95% CI: 1.60–7.11, *p* = 0.001). HIV status remained significant after correcting for multiple comparisons (Supplementary Table S6).

**Table 5:** Multivariable analyses of factors associated with oral, anal and both sites high-risk HPV.

Variables	Oral hrHPV		Anal hrHPV		Oral and Anal hrHPV	
	Crude OR	Adjusted OR	Crude OR	Adjusted OR	Crude OR	Adjusted OR
Primary Explanatory Variable						
Gender	***p* < 0.001**	***p* = 0.011**	***p* < 0.001**	***p* < 0.001**	***p* < 0.001**	***p* = 0.002**
FSW	1	1	1	1	1	1
FGP	0.67 (0.41–1.10)	1.70 (0.62–4.63)	0.42 (0.30–0.58)	0.83 (0.43–1.59)	0.55 (0.31–0.96)	2.90 (0.87–9.69)
Male	0.21 (0.11–0.42)	0.43 (0.15–1.24)	0.05 (0.03–0.08)	0.06 (0.03–0.13)	0.06 (0.01–0.25)	0.17 (0.03–0.95)
Socio-demographic Characteristics						
Age		*p* = 0.868		***p* = 0.043**		*p* = 0.573
18–24 years		1		1		1
25–34 Years		1.09 (0.58–2.04)		0.63 (0.41–0.96)		0.89 (0.42–1.88)
35–45 Years		0.92 (0.46–1.86)		0.59 (0.37–0.93)		0.64 (0.27–1.52)
Education		*p* = 0.484		*p* = 0.243		*p* = 0.419
No Education		1		1		1
Primary		0.42 (0.13–1.38)		2.11 (0.79–5.63)		0.40 (0.10–1.57)
Secondary		0.62 (0.21–1.79)		2.52 (1.00–6.37)		0.66 (0.20–2.14)
Tertiary		0.69 (0.21–2.35)		2.49 (0.90–6.88)		0.92 (0.23–3.65)
Occupation		*p* = 0.746		*p* = 0.549		*p* = 0.909
No current paid job		1		1		1
Unskilled		0.42 (0.13–1.38)		0.45 (0.14–1.46)		0.58 (0.06–5.60)
Semi-Skilled		0.62 (0.21–1.79)		0.81 (0.51–1.29)		0.76 (0.34–1.72)
Skilled		0.69 (0.21–2.35)		0.68 (0.25–1.85)		0.67 (0.07–6.38)
Monthly Income		*p* = 0.913		***p* = 0.029**		*p* = 0.507
No Income		1		1		1
1–10,000 N (1–28USD)		1.37 (0.37–5.07)		1.22 (0.55–2.71)		0.63 (0.14–2.87)
10,001–20,000 N (> 28–56USD)		1.47 (0.40–5.49)		0.82 (0.36–1.85)		1.38 (0.32–5.93)
>20,000 N (> 56USD)		1.60 (0.43–6.05)		1.57 (0.68–3.58)		1.35 (0.31–5.98)
Marital Status		*p* = 0.458		*p* = 0.717		*p* = 0.417
Single		1		1		1
Married		1.15 (0.55–2.39)		0.83 (0.52–1.32)		0.65 (0.26–1.62)
Divorced/Widow		1.50 (0.79–2.87)		0.94 (0.59–1.48)		1.37 (0.66–2.83)
Partner has another sexual partner		*p* = 0.329		*p* = 0.794		*p* = 0.107
Don't know		1		1		1
No		0.61 (0.32–1.17)		0.89 (0.58–1.38)		0.45 (0.20–0.99)
Yes		0.81 (0.44–1.49)		0.87 (0.56–1.33)		0.58 (0.28–1.20)
Study Setting		*p* = 0.082		***p* = 0.043**		*p* = 0.610
Urban		1		1		1
Rural		0.57 (0.30–1.07)		0.66 (0.45–0.99)		0.82 (0.39–1.75)
Social and Lifestyle and Sexual Characteristics						
Ever given oral sex		*p* = 0.689				*p* = 0.476
No		1		-		1
Yes		1.15 (0.59–2.24)		-		1.33 (0.60–2.94)
Ever had anal sex				*p* = 0.962		***p* = 0.025**
No		-		1		1
Yes		-		1.03 (0.30–3.53)		5.51 (1.24–24.44)
Ever drank alcohol		*p* = 0.852		*p* = 0.223		*p* = 0.868
No		1		1		1
Yes		0.95 (0.55–1.65)		0.79 (0.54–1.16)		0.94 (0.48–1.84)
Ever smoked tobacco or cigarette		*p* = 0.831		***p* = 0.012**		*p* = 0.217
No		1		1		1
Yes		1.08 (0.55–2.11)		1.82 (1.14–2.91)		1.64 (0.74–3.57)
Biological Characteristics						
Ever had any STI		*p* = 0.865		*p* = 0.531		*p* = 0.975
No		1		1		1
Yes		1.07 (0.51–2.25)		1.17 (0.72–1.91)		1.01 (0.43–2.38)
Diagnosed of HIV		***p* = 0.001**		***p* < 0.001**		***p* < 0.001**
No		1		1		1
Yes		3.37 (1.60–7.11)		4.44 (2.04–9.67)		5.40 (2.42–12.04)

hrHPV-16, 18, 31, 33, 35, 39, 45, 51, 52, 56, 58, 59, 66, 68.

The bold values in the table indicate significant *p*-value.

Again, sex of participants was associated with odds of anal hrHPV with male participants (cOR = 0.05, 95% CI: 0.03–0.08) having lower odds relative to FSWs and FGP (cOR = 0.42, 95% CI: 0.30–0.58). The pattern of the odds of anal hrHPV remains the same in the adjusted model. Sex of participants, age group, monthly income, study settings, history of smoking or tobacco use, and HIV status were significantly associated with the odds of anal hrHPV. The odds of having anal hrHPV reduces with increasing age group, with participants aged 35 years and above (aOR = 0.59, 95% CI: 0.37–0.93) having lower odds compared to those aged 18–24 years. Participants living in rural settings had lower odds of anal hrHPV compared to those living in urban settings. The odds of anal hrHPV was higher among participants with history of cigarette smoking or tobacco use (aOR = 1.82, 95% CI: 1.14–2.91) compared to those with no history of cigarette smoking or tobacco use. The odds of anal hrHPV were 4.44 times higher among HIV-positive participants (95% CI: 2.04–9.67, *p* < 0.001). All the significant variables in the adjusted model, except HIV status, lost statistical significance after correcting for multiple hypotheses (Supplementary Table S6).

In the crude model, odds of hrHPV in both oral and anal sites was lower among males (cOR = 0.06, 95% CI: 0.01–0.25) compared to FSW and FGP (cOR = 0.55, 95% CI: 0.31–0.96). After adjusting for socio-economic, lifestyle, and biological characteristics, FGP (aOR = 2.90, 95% CI: 0.87–9.69) had higher odds of hrHPV in oral and anal sites compared to FSWs and males (aOR = 0.17, 95% CI: 0.03–0.95). History of anal sex (aOR = 5.51, 95% CI: 1.24–24.44), and HIV status (aOR = 5.40, 95% CI: 2.42–12.04) were significantly associated with the odds of hrHPV at both sites. HIV status remains significant after correcting for multiple hypotheses (Supplementary Table S6).

## 4 Discussion

This study investigated the sex differences in the prevalence of oral and anal hrHPV infections, as well as their associated factors among sexually active individuals in Ibadan, Nigeria. The findings showed a higher prevalence of oral and anal hrHPV infections among FGP (10.5% and 39.3%, respectively) and FSWs (14.9% and 60.8%, respectively) compared to males (3.6% and 6.7%, respectively). The odds of oral hrHPV infection was significantly associated with sex and HIV status, while the odds of anal hrHPV infection was associated with sex, age, monthly income, location, history of smoking, and HIV status. Males and females exhibit distinct patterns in both the prevalence and risk factors for oral and anal hrHPV infections.

As the largest study of HPV infection among Nigeria's sexually active population, this study showed a similar prevalence rate similar to other studies conducted in SSA (21, 22) but a distinct difference in the prevalence of oral hrHPV infection when compared to community-based studies conducted in the USA and other Western countries (11, 23, 24). We found that the prevalence and odds of oral hrHPV infection were significantly higher among FGP and FSWs. This sex disparity persisted even after adjusting for sociodemographic status, sexual behavior, social lifestyle, and biological characteristics. These findings suggest that the higher prevalence of oral hrHPV infection in females may be primarily driven by biological differences between the sexes, rather than behavioral factors that might increase males' exposure to oral HPV infection.

The higher prevalence of oral hrHPV among females compared to males may also be influenced by cultural norms specific to Nigeria and Sub-Saharan Africa. In these regions, there is a cultural expectation that females should be submissive in relationships, often prioritizing their partner's sexual desires. This dynamic may lead to an imbalance in sexual practices, where males are more likely to receive oral sex but less likely to perform it on their female partners. Religious, cultural, and traditional beliefs often discourage African men from engaging in cunnilingus, while fellatio is more commonly practiced. This pattern has been observed in research from Southeast Nigeria, where fellatio was reported more frequently than cunnilingus (25). These cultural practices may explain the higher exposure to oral hrHPV among females, contributing to the observed sex disparity in the infection rates.

The significant association regardless of the subgroup of females whose current sexual partners have other sexual partners and the prevalence of hrHPV at anal site and at both site (oral and anal) in our study further buttresses the gender-based power imbalance as a factor contributing to the higher prevalence of hrHPV in females than males. In Nigeria and SSA, the cultural norm that a man can marry more than one wife limits women's ability to inquire about or address their partner's sexual behaviors and negotiate safer sexual practices. Women who are unaware of their partner's sexual escapade may lack the knowledge or awareness necessary to take protective measures, such as consistent condom use or regular HPV screening, thereby placing them at higher risk of hrHPV.

Numerous studies have established the association of oral and oropharyngeal HPV infections to various factors, including age, open-mouth kissing, HIV infection, oral sex practices, and abnormalities of the oral mucosa (26–29). Consistent with these findings, our study observed a significant association between oral hrHPV infection among females and their history of oral sex. This supports the established association between oral sexual behaviors and an increased risk of oral HPV infection. Previous research has also reported an association between oral HPV infection, smoking, and alcohol consumption (30–32). In our study, participants who reported alcohol consumption among females had nearly two times the prevalence of oral hrHPV infection compared to those who did not. Conversely, we found a lower prevalence of oral HPV infection among those who reported smoking tobacco or cigarettes. This suggests that, despite variations in smoking and sexual behavior, alcohol consumption may not be a straightforward risk factor for oral hrHPV infection in our study population.

Contrary to the trends observed in oral hrHPV infection, our findings align with previous research indicating that anal hrHPV infection is more prevalent among females than males (33–35). Studies on anal HPV prevalence among men who have sex with women report an infection rate of approximately 12%, which is about half of what is found in women (33). Consistent with most research on genital HPV (36, 37), our study also demonstrates a decline in the prevalence of anal hrHPV infection among FGP as they age. However, our findings differ from reports suggesting that the prevalence of anal hrHPV infection increases with age among men who have sex with women (38). Interestingly, the percentage of both males and females in our study who reported engaging in anal sex was remarkably low. This observation suggests that nonsexual transmission routes, such as auto-inoculation, passive touch, or even women's frequent use of douches, which might transfer the virus from the vaginal area to the anus, could play a significant role in the spread of anal HPV in this population. These findings highlight the complexity of HPV transmission and suggest that additional factors beyond sexual behavior may contribute to the higher rates of anal hrHPV infection observed among FGP.

Despite the low percentage of males and females reporting anal sex, the odds of hrHPV at both the oral and anal sites were significantly higher in individuals with a history of anal sex compared to those that had never experienced anal sex. Although the prevalence of anal sex in this study population is low, individuals who engaged in it had an increased risk of hrHPV infection in both sites. However, the low prevalence of anal sex could limit the generalizability of this association. Further research with larger sample sizes and targeted populations is needed to confirm these findings and to better understand the underlying mechanisms driving this relationship.

Given the established association between oral sex history and the prevalence of hrHPV infection, we observed a lower prevalence of hrHPV among females and males living in rural areas. This finding can be explained by the sexual behaviors prevalent in these communities. Individuals in rural settlements are more likely to engage predominantly in vaginal intercourse, with oral and anal sex being less common (39, 40). Cultural and social norms in rural areas often discourage oral and anal sex, which might explain the reduced transmission rates of hrHPV through these routes. Also, limited access to sexual health education and services in rural areas may also influence sexual practices, leading to lower adoption of behaviors such as oral sex, which are more commonly associated with hrHPV transmission. This pattern contrasts with urban populations, where there may be greater exposure to diverse sexual practices, including oral and anal sex, contributing to higher hrHPV prevalence. The lower prevalence in rural areas underscores the importance of understanding how cultural, social, and geographic factors shape sexual behaviors and, consequently, the transmission of hrHPV.

There is a paucity of studies on the prevalence of oral and anal HPV that include both males and females in the general population within SSA. To the best of our knowledge, this is the first study to examine and compare the prevalence of HPV in both oral and anal sites, and each site independently, between the sexes in the general population in Nigeria and SSA. In Nigeria, a previous smaller study reported the prevalence of oral HPV among 104 males and 127 females attending dental clinics, with a higher prevalence observed in males (11.5%) compared to females (7.1%) (41). However, this study only included dental clinic patients and does not represent findings from the general population. Our study expands this evidence by utilizing data from the general population, which provides a more representative understanding of HPV prevalence across sexes and anatomical sites. This broader context enables more insights into HPV epidemiology in Nigeria and SSA and improves the global understanding of HPV burden, particularly in countries with similar income categories.

We acknowledge that self-reported data may be skewed by social desirability bias, especially when it comes to delicate subjects like oral and anal sexual behaviors. It is plausible that some participants might have underreported. This underreporting can lead to misclassification, weaken the observed associations between sexual behaviors and hrHPV infection, and result in potentially biased estimates of risk. A previous qualitative study showed that males and FSWs, relative to FGP, were more likely to report having oral and anal sex (42). In the current study, however, steps were taken to reduce the possible influence of social desirability bias. Sex-matched research assistants conducted live, in-person interviews to collect the data. This approach minimizes the possibility of potential bias from participant sharing their sexual experience with opposite sex especially among women in a relatively conservative environment. The face-to-face interview promotes opportunities for participants to seek clarification and interviewer support. We believe that the reporting of oral and anal sex in this dataset is not significantly impacted by social desirability bias, despite the possibility that it exists to some degree. The study's inability to collect detailed household information for participants restricted our ability to account for potential clustering effects of HPV infections and related risk factors within community settings. The cross-sectional design of this study makes it impossible to assess causality and the participants' timing of HPV acquisition. Despite these limitations, this study provided the first and unique information on sex differences in the burden of hrHPV in males and females from population data in Nigeria and in West Africa.

In conclusion, this study showed that oral and anal hrHPV infections are more prevalent in females than males after adjusting for socio-economic and lifestyle characteristics. However, this will not diminish the importance of focusing on HPV prevention strategies in males. We recommend longitudinal studies to further explore this hypothesis to understand biological mechanisms contributing to these disparities, which could inform targeted prevention strategies and improve HPV-related health outcomes.

## Data Availability

The raw data supporting the conclusions of this article will be made available by the authors, without undue reservation.
