# Supplementary material for: Sex differences in the prevalence of high-risk oral and anal human papillomavirus infections among heterosexually active populations in Ibadan, Nigeria
Source: Front Reprod Health. 2025 Aug 18;7:1570984. doi: 10.3389/frph.2025.1570984 (PMC12399633; doi:10.3389/frph.2025.1570984)
Supplement: Supplementary file 1 [file Table1.docx]

**Supplementary Tables**

Supplementary Table 1: Prevalence of oral, anal and both sites high-risk HPV by sex irrespective of female subgroups

| **Variable** | **Sex** | | **p-value** |
| --- | --- | --- | --- |
|  | **Female**  **n(%)** | **Male**  **n(%)** |  |
| **Oral hrHPV** |  |  | < 0.001 |
| No | 496 (87.3) | 297 (96.4) |  |
| Yes | 72 (12.7) | 11 (3.6) |  |
| **Anal hrHPV** |  |  | < 0.001 |
| No | 306 (49.8) | 223 (93.3) |  |
| Yes | 308 (50.2) | 16 (6.7) |  |
| **Oral and Anal hrHPV** |  |  | < 0.001 |
| No | 501 (89.8) | 229 (99.1) |  |
| Yes | 57 (10.2) | 2 (0.9) |  |

Supplementary Table 2: Characteristics and prevalence of oral high-risk HPV among females irrespective of subgroup

| **Explanatory Variable** | **Female^1^**  **Oral hrHPV** | | **p-value** |
| --- | --- | --- | --- |
|  | **Yes (N = 72)**  **n (Col %)** | **No (N = 496)**  **n (Col %)** |  |
| **Socio-demographic Characteristics** | | | |
| **Age** |  |  | 0.381 |
| 18 – 24 years | 21 (29.2) | 136 (27.4) |  |
| 25-34 Years | 35 (48.6) | 211 (42.5) |  |
| 35-45 Years | 16 (22.2) | 149 (30.0) |  |
| **Education** |  |  | 0.199 |
| None | 6 (8.3) | 17 (3.4) |  |
| Primary | 11 (15.3) | 102 (20.6) |  |
| Secondary | 43 (59.7) | 299 (60.3) |  |
| Tertiary | 12 (16.7) | 78 (15.7) |  |
| **Occupation** |  |  | 0.579 |
| No current paid job | 38 (52.8) | 219 (44.2) |  |
| Unskilled | 2 (2.8) | 15 (3.0) |  |
| Semi-Skilled | 31 (43.0) | 245 (49.4) |  |
| Skilled | 1 (1.4) | 17 (3.4) |  |
| **Monthly Income** |  |  | 0.665 |
| No income | 3 (4.2) | 34 (6.8) |  |
| 1–10,000 N (1–28USD) | 13 (18.0) | 113 (22.8) |  |
| 10,001–20,000 N (> 28–56USD) | 20 (27.8) | 130 (26.2) |  |
| > 20,000 N (> 56USD) | 36 (50.0) | 219 (44.2) |  |
| Marital Status |  |  | 0.122 |
| Single | 28 (38.9) | 200 (40.3) |  |
| Married | 20 (27.8) | 182 (36.7) |  |
| Divorced/Widow | 24 (33.3) | 114 (23.0) |  |
| Partner has another sexual partner (N = 557) |  |  | 0.095 |
| Don’t know | 29 (40.9) | 152 (31.3) |  |
| No | 19 (26.8) | 193 (39.7) |  |
| Yes | 23 (32.4) | 141 (29.0) |  |
| **Study Setting** |  |  | 0.132 |
| Urban | 56 (77.8) | 341 (68.7) |  |
| Rural | 16 (22.2) | 155 (31.3) |  |
| **Sexual Behaviours Characteristics** | | | |
| **Age at first gave oral sex** (mean (SD)) | 24.8 (2.4) | 26.2 (0.8) |  |
| **Age at first received oral sex** (mean (SD)) | 27.6 (6.6) | 26.6 (6.4) |  |
| **Ever given oral sex** |  |  | 0.234 |
| Yes | 14 (19.4) | 70 (14.1) |  |
| No | 58 (80.6) | 426 (85.9) |  |
| **Ever received oral sex** |  |  | **0.050** |
| Yes | 26 (36.1) | 125 (25.2) |  |
| No | 46 (63.9) | 371 (74.8) |  |
| Number of oral sex partners (median (IQR)) | 1 (1) | 1 (0) |  |
| Condom/barrier use during last oral sex (N = 84) |  |  | 0.341 |
| Yes | 0 | 8 (11.4) |  |
| No | 14 (100) | 62 (88.6) |  |
| **Social and Lifestyle Characteristics** | | | |
| Ever drank alcohol |  |  | 0.332 |
| Yes | 50 (69.4) | 371 (74.8) |  |
| No | 22 (30.6) | 125 (25.2) |  |
| Ever taken any illicit drugs |  |  | - |
| Yes | 0 | 0 |  |
| No | 72 (100) | 496 (100) |  |
| **Ever smoked tobacco or cigarette** |  |  | 0.180 |
| Yes | 15 (20.8) | 73 (14.7) |  |
| No | 57 (79.2) | 423 (85.3) |  |
| **Biological Characteristics** | | | |
| **Ever had any STI** |  |  | 0.792 |
| No | 61 (84.7) | 426 (85.9) |  |
| Yes | 11 (15.3) | 70 (14.1) |  |
| **Diagnosed of HIV** |  |  | **<0.001** |
| Yes | 14 (19.4) | 31 (6.3) |  |
| No | 58 (80.6) | 465 (93.7) |  |

*hrHPV-16,18,31,33,35,39,45,51,52,56,58,59,66,68; 1 – 57 participants with invalid sample*

Supplementary Table 3: Characteristics and prevalence of anal high-risk HPV among females irrespective of subgroup

| **Explanatory Variable** | **Female^1^**  **Anal hrHPV** | | **p-value** |
| --- | --- | --- | --- |
|  | **Yes (N = 308)**  **n (Col %)** | **No (N = 306)**  **n (Col %)** |  |
| **Socio-demographic Characteristics** | | | |
| **Age** |  |  | 0.124 |
| 18 – 24 years | 97 (31.5) | 78 (25.5) |  |
| 25-34 Years | 135 (43.8) | 133 (43.5) |  |
| 35-45 Years | 76 (24.7) | 95 (31.1) |  |
| **Education** |  |  | 0.659 |
| None | 12 (3.9) | 14 (4.6) |  |
| Primary | 56 (18.2) | 65 (21.2) |  |
| Secondary | 193 (62.7) | 177 (57.8) |  |
| Tertiary | 47 (15.3) | 50 (16.3) |  |
| **Occupation** |  |  | **<0.001** |
| No current paid job | 169 (54.9) | 111 (36.3) |  |
| Unskilled | 5 (1.6) | 13 (4.3) |  |
| Semi-Skilled | 127 (41.2) | 168 (54.9) |  |
| Skilled | 7 (2.3) | 14 (4.6) |  |
| **Monthly Income** |  |  | **<0.001** |
| No income | 18 (5.8) | 20 (6.5) |  |
| 1–10,000 N (1–28USD) | 57 (18.5) | 80 (26.1) |  |
| 10,001–20,000 N (> 28–56USD) | 67 (21.8) | 95 (31.1) |  |
| > 20,000 N (> 56USD) | 166 (53.9) | 111 (36.3) |  |
| Marital Status |  |  | **<0.001** |
| Single | 136 (44.2) | 112 (36.6) |  |
| Married | 83 (27.0) | 132 (43.1) |  |
| Divorced/Widow | 89 (28.9) | 62 (20.3) |  |
| Partner has another sexual partner (N = 602) |  |  | **0.012** |
| Don’t know | 112 (37.2) | 82 (27.2) |  |
| No | 99 (32.9) | 130 (43.2) |  |
| Yes | 90 (29.9) | 89 (29.6) |  |
| **Study Setting** |  |  | **<0.001** |
| Urban | 238 (77.3) | 192 (62.8) |  |
| Rural | 70 (22.7) | 114 (37.3) |  |
| **Sexual Behavioral Characteristics** | | | |
| **Age at first anal sex** (mean (SD)) | 24.0 (2.6) | 21.7 (0.9) | 0.557 |
| **Ever had anal sex** |  |  | 0.505 |
| Yes | 6 (2.0) | 3 (1.0) |  |
| No | 302 (98.1) | 303 (99.0) |  |
| Number of anal sex partners (median (IQR)) | 1 (0) | 1 (6) |  |
| Condom/barrier use during last anal sex (N = 9) |  |  | 0.635 |
| Yes | 3 (50.0) | 2(66.7) |  |
| No | 3 (50.0) | 1 (33.3) |  |
| **Social and Lifestyle Characteristics** | | | |
| Ever drank alcohol |  |  | 0.089 |
| Yes | 219 (71.1) | 236 (77.1) |  |
| No | 89 (28.9) | 70 (22.9) |  |
| Ever taken any illicit drugs |  |  | - |
| Yes | 0 | 0 |  |
| No | 308 (100) | 306 (100) |  |
| **Ever smoked tobacco or cigarette** |  |  | **<0.001** |
| Yes | 70 (22.7) | 35 (11.4) |  |
| No | 238 (77.3) | 271 (88.6) |  |
| **Biological Characteristics** | | | |
| **Ever had any STI** |  |  | 0.585 |
| No | 262 (85.1) | 265 (86.6) |  |
| Yes | 46 (14.9) | 41 (13.4) |  |
| **Diagnosed of HIV** |  |  | **<0.001** |
| Yes | 41 (13.3) | 9 (2.9) |  |
| No | 267 (86.7) | 297 (97.1) |  |

*hrHPV-16,18,31,33,35,39,45,51,52,56,58,59,66,68; 1 – 11 participants with invalid sample*

Supplementary Table 4: Characteristics and prevalence of high-risk HPV in both oral and anal sites among females irrespective of subgroup

| **Explanatory Variable** | **Female^1^**  **Oral and Anal hrHPV** | | **p-value** |
| --- | --- | --- | --- |
|  | **Yes (N = 57)**  **n (C**ol **%)** | **No (N = 501)**  **n (C**ol **%)** |  |
| **Socio-demographic Characteristics** | | | |
| **Age** |  |  | 0.256 |
| 18 – 24 years | 18 (31.6) | 138 (27.5) |  |
| 25-34 Years | 28 (49.1) | 214 (42.7) |  |
| 35-45 Years | 11 (19.3) | 149 (29.7) |  |
| **Education** |  |  | **0.032** |
| None | 6 (10.5) | 16 (3.2) |  |
| Primary | 7 (12.3) | 106 (21.2) |  |
| Secondary | 33 (57.9) | 302 (60.3) |  |
| Tertiary | 11 (19.3) | 77 (15.4) |  |
| **Occupation** |  |  | 0.282 |
| No current paid job | 33 (57.9) | 221 (44.1) |  |
| Unskilled | 1 (1.8) | 16 (3.2) |  |
| Semi-Skilled | 22 (38.6) | 247 (49.3) |  |
| Skilled | 1 (1.8) | 17 (3.4) |  |
| **Monthly Income** |  |  | 0.109 |
| No income | 3 (5.3) | 34 (6.8) |  |
| 1–10,000 N (1–28USD) | 6 (10.5) | 117 (23.4) |  |
| 10,001–20,000 N (> 28–56USD) | 16 (28.1) | 130 (26.0) |  |
| > 20,000 N (> 56USD) | 32 (56.1) | 220 (43.9) |  |
| Marital Status |  |  | **0.034** |
| Single | 25 (43.9) | 201 (40.1) |  |
| Married | 12 (21.1) | 184 (36.7) |  |
| Divorced/Widow | 20 (35.1) | 116 (23.2) |  |
| Partner has another sexual partner (N = 547) |  |  | **0.036** |
| Don’t know | 26 (46.4) | 151 (30.8) |  |
| No | 14 (25.0) | 194 (39.5) |  |
| Yes | 16 (28.6) | 146 (29.7) |  |
| **Study Setting** |  |  | 0.205 |
| Urban | 44 (77.2) | 346 (69.1) |  |
| Rural | 13 (22.8) | 155 (30.9) |  |
| **Sexual Behaviors Characteristics** | | | |
| **Age at first gave oral sex** (mean (SD)) | 23.2 (2.0) | 26.6 (0.9) | 0.158 |
| **Age at first received oral sex** (mean (SD)) | 26.6 (1.3) | 26.9 (0.6) | 0.796 |
| **Ever given oral sex** |  |  | 0.068 |
| Yes | 13 (22.8) | 69 (13.8) |  |
| No | 44 (77.2) | 432 (86.3) |  |
| **Ever received oral sex** |  |  | 0.058 |
| Yes | 21 (36.8) | 126 (25.2) |  |
| No | 36 (63.2) | 375 (74.9) |  |
| Number of oral sex partners (median (IQR)) | 1 (1) | 1 (0) |  |
| **Ever had anal sex** |  |  | **0.005** |
| Yes | 4 (7.0) | 4 (0.8) |  |
| No | 53 (93.0) | 497 (99.2) |  |
| **Age at first anal sex** (mean (SD)) | 22.8 (1.7) | 24.0 (3.8) | 0.774 |
| Number of anal sex partners (median (IQR)) | 1 (2) | 1(0) |  |
| Condom/barrier use during last oral sex (N = 82) |  |  | 0.344 |
| Yes | 0 | 8 (11.6) |  |
| No | 13/13 (100) | 61 (88.4) |  |
| **Social and Lifestyle Characteristics** | | | |
| Ever drank alcohol |  |  | 0.310 |
| Yes | 39 (68.4) | 374 (74.7) |  |
| No | 18 (31.6) | 127 (25.4) |  |
| Ever taken any illicit drugs |  |  |  |
| Yes | 0 | 0 |  |
| No | 57 (100) | 501 (100) |  |
| **Ever smoked tobacco or cigarette** |  |  | **0.016** |
| Yes | 15 (26.3) | 71 (14.2) |  |
| No | 42 (73.7) | 430 (85.8) |  |
| **Biological Characteristics** | | | |
| **Ever had any STI** |  |  | 0.709 |
| No | 48 (84.2) | 431 (86.0) |  |
| Yes | 9 (15.8) | 70 (14.0) |  |
| **Diagnosed of HIV** |  |  | **<0.001** |
| Yes | 14 (24.6) | 31 (6.2) |  |
| No | 43 (75.4) | 470 (93.8) |  |

*hrHPV-16,18,31,33,35,39,45,51,52,56,58,59,66,68; 1 – 67 participants with invalid sample*

Supplementary Table 5: Post-Hoc Power Analysis of Pairwise Comparisons of high-risk HPV Prevalence

| **Multiple pairwise comparisons** | **Power** |
| --- | --- |
| **Oral hrHPV** |  |
| Male vs. FGP | 90.99% |
| Male vs. FSW | 99.76% |
| FGP vs. FSW | 35.03% |
| **Anal hrHPV** |  |
| Male vs. FGP | >99.99% |
| Male vs. FSW | >99.99% |
| FGP vs. FSW | 99.97% |
| **Both site** |  |
| Male vs. FGP | 96.10% |
| Male vs. FSW | 99.98% |
| FGP vs. FSW | 57.27% |

Supplementary Table 6: Multivariable analyses of factors associated with oral, anal and both sites high-risk HPV, with Hommel’s adjusted p-values

| **Variables** | **Oral hrHPV** | | **Anal hrHPV** | | **Oral and Anal hrHPV** | |
| --- | --- | --- | --- | --- | --- | --- |
|  | **Crude OR** | **Adjusted OR** | **Crude OR** | **Adjusted OR** | **Crude OR** | **Adjusted OR** |
| **Primary Explanatory Variable** | | | | | | |
| **Gender** | **p < 0.001** | **p = 0.011** | **p < 0.001** | **p < 0.001** | **p < 0.001** | **p = 0.002** |
| FSW | 1 | 1 | 1 | 1 | 1 | 1 |
| FGP | 0.67 (0.41 – 1.10) | 1.70 (0.62 – 4.63) | 0.42 (0.30 – 0.58) | 0.83 (0.43 – 1.59) | 0.55 (0.31 – 0.96) | 2.90 (0.87 – 9.69) |
| Male | 0.21 (0.11 – 0.42) | 0.43 (0.15 – 1.24) | 0.05 (0.03 – 0.08) | 0.06 (0.03 – 0.13) | 0.06 (0.01 – 0.25) | 0.17 (0.03 – 0.95) |
| **Socio-demographic Characteristics** | | | | | | |
| **Age** |  | p = 0.913 |  | p = 0.344 |  | p = 0.975 |
| 18 – 24 years |  | 1 |  | 1 |  | 1 |
| 25-34 Years |  | 1.09 (0.58 – 2.04) |  | 0.63 (0.41 – 0.96) |  | 0.89 (0.42 – 1.88) |
| 35-45 Years |  | 0.92 (0.46 – 1.86) |  | 0.59 (0.37 – 0.93) |  | 0.64 (0.27 – 1.52) |
| **Education** |  | p = 0.913 |  | p = 0.962 |  | p = 0.975 |
| No Education |  | 1 |  | 1 |  | 1 |
| Primary |  | 0.42 (0.13 – 1.38) |  | 2.11 (0.79 – 5.63) |  | 0.40 (0.10 – 1.57) |
| Secondary |  | 0.62 (0.21 – 1.79) |  | 2.52 (1.00 – 6.37) |  | 0.66 (0.20 – 2.14) |
| Tertiary |  | 0.69 (0.21 – 2.35) |  | 2.49 (0.90 – 6.88) |  | 0.92 (0.23 – 3.65) |
| **Occupation** |  | p = 0.913 |  | p = 0.962 |  | p = 0.975 |
| No current paid job |  | 1 |  | 1 |  | 1 |
| Unskilled |  | 0.42 (0.13 – 1.38) |  | 0.45 (0.14 – 1.46) |  | 0.58 (0.06 – 5.60) |
| Semi-Skilled |  | 0.62 (0.21 – 1.79) |  | 0.81 (0.51 – 1.29) |  | 0.76 (0.34 – 1.72) |
| Skilled |  | 0.69 (0.21 – 2.35) |  | 0.68 (0.25 – 1.85) |  | 0.67 (0.07 – 6.38) |
| **Monthly Income** |  | p = 0.913 |  | p = 0.232 |  | p = 0.975 |
| No Income |  | 1 |  | 1 |  | 1 |
| 1–10,000 N (1–28USD) |  | 1.37 (0.37 – 5.07) |  | 1.22 (0.55 – 2.71) |  | 0.63 (0.14 – 2.87) |
| 10,001–20,000 N (> 28–56USD) |  | 1.47 (0.40 – 5.49) |  | 0.82 (0.36 – 1.85) |  | 1.38 (0.32 – 5.93) |
| > 20,000 N (> 56USD) |  | 1.60 (0.43 – 6.05) |  | 1.57 (0.68 – 3.58) |  | 1.35 (0.31 – 5.98) |
| Marital Status |  | p = 0.913 |  | p = 0.962 |  | p = 0.975 |
| Single |  | 1 |  | 1 |  | 1 |
| Married |  | 1.15 (0.55 – 2.39) |  | 0.83 (0.52 – 1.32) |  | 0.65 (0.26 – 1.62) |
| Divorced/Widow |  | 1.50 (0.79 – 2.87) |  | 0.94 (0.59 – 1.48) |  | 1.37 (0.66 – 2.83) |
| Partner has another sexual partner |  | p = 0.913 |  | p = 0.962 |  | p = 0.915 |
| Don’t know |  | 1 |  | 1 |  | 1 |
| No |  | 0.61 (0.32 – 1.17) |  | 0.89 (0.58 – 1.38) |  | 0.45 (0.20 – 0.99) |
| Yes |  | 0.81 (0.44 – 1.49) |  | 0.87 (0.56 – 1.33) |  | 0.58 (0.28 – 1.20) |
| **Study Setting** |  | p = 0.902 |  | p = 0.344 |  | p = 0.975 |
| Urban |  | 1 |  | 1 |  | 1 |
| Rural |  | 0.57 (0.30 – 1.07) |  | 0.66 (0.45 – 0.99) |  | 0.82 (0.39 – 1.75) |
| **Social and Lifestyle Characteristics** | | | | | | |
| Ever give oral sex |  | p = 0.913 |  |  |  | p = 0.975 |
| No |  | 1 |  | - |  | 1 |
| Yes |  | 1.15 (0.59 – 2.24) |  | - |  | 1.33 (0.60 – 2.94) |
| Ever had anal sex |  |  |  | p = 0.962 |  | p = 0.300 |
| No |  | - |  | 1 |  | 1 |
| Yes |  | - |  | 1.03 (0.30 – 3.53) |  | 5.51 (1.24 – 24.44) |
| Ever drank alcohol |  | p = 0.913 |  | p = 0.962 |  | p = 0.975 |
| No |  | 1 |  | 1 |  | 1 |
| Yes |  | 0.95 (0.55 – 1.65) |  | 0.79 (0.54 – 1.16) |  | 0.94 (0.48 – 1.84) |
| **Ever smoked tobacco or cigarette** |  | p = 0.913 |  | p = 0.120 |  | p = 0.975 |
| No |  | 1 |  | 1 |  | 1 |
| Yes |  | 1.08 (0.55 – 2.11) |  | 1.82 (1.14 – 2.91) |  | 1.64 (0.74 – 3.57) |
| **Biological Characteristics** | | | | | | |
| **Ever had any STI** |  | p = 0.913 |  | p = 0.962 |  | p = 0.975 |
| No |  | 1 |  | 1 |  | 1 |
| Yes |  | 1.07 (0.51 – 2.25) |  | 1.17 (0.72 – 1.91) |  | 1.01 (0.43 – 2.38) |
| **Diagnosed of HIV** |  | **p = 0.012** |  | **p = 0.012** |  | **p = 0.013** |
| No |  | 1 |  | 1 |  | 1 |
| Yes |  | 3.37 (1.60 – 7.11) |  | 4.44 (2.04 – 9.67) |  | 5.40 (2.42 – 12.04) |

hrHPV-16,18,31,33,35,39,45,51,52,56,58,59,66,68

**Supplementary Figures**

**Study population (N=310)**

**Ibadan North LGA**

**450** females listed

**Akinyele LGA**

**480** females listed

**304** were randomly selected and visited at home

**297** were randomly selected and visited at home

**177 (58%)** enrolled and invited to the clinic to participate

**159** **(54%)** enrolled and invited to the clinic to participate

**127 (42%)** were not found at home after 3 visits

**138 (46%)** were not found at home after 3 visits

**157 (89%) participated in the study**

**153 (96%) participated in the study**

**7 (4%)** refused participation

**13 (7%)** ineligible participants

**2 (1%)** refused participation

**4 (3%)** ineligible participants

**930** **Females**

Supplementary Figure 1: FGP enrolment flow chart for SHINI study

Supplementary Figure 2: Male participant enrolment for SHINI study

**Study population (N=316)**

**866 Sexually active men**

**Ibadan North LGA**

**502** men listed

**Akinyele LGA**

364 men listed

**308** were randomly selected & visited at home

**285** were randomly selected & visited at home

**184** (60%) enrolled & invited to the clinic

**154** (54%) enrolled & invited to the clinic

**163** (89%) participated in the study

**153** (99%) participated in the study

**124** (40%) were not at home after 3 visitations

**131** (46%) were not at home after 3 visitations

**8** (4%) refused to participate

**13** (7%) were ineligible

**1** (<1%) refused to participate

Supplementary Figure 3: FSW enrolment flow chart for the SHINI study

**31 brothels in 6 local government areas** listed and invited to participate

**28 brothels** participated in the survey

**344 FSWs** listed in **28** brothels

**319 (93%) FSWs** randomly selected and enrolled

**315 (99%) FSWs** participated

**2 brothels** closed

**1 brothel** declined

**4 (1%) FSWs** declined participation
